# Delayed treatment in breast cancer patients during the COVID-19 pandemic: a population health information research infrastructure (PHIRI) case study

**DOI:** 10.1093/eurpub/ckae038

**Published:** 2024-07-01

**Authors:** Francisco Estupiñán-Romero, Santiago Royo-Sierra, Javier González-Galindo, Natalia Martínez-Lizaga, Petronille Bogaert, Nienke Schutte, Liesbet Van Eycken, Nancy Van Damme, Kris Henau, Ronan A Lyons, Sarah J Aldridge, Andrea Faragalli, Flavia Carle, Rosaria Gesuita, Luigi Palmieri, Jānis Misiņš, Martin Thiβen, Enrique Bernal-Delgado

**Affiliations:** Data Science for Health Services and Policy Research, Health Science Institute in Aragon (IACS), Aragon, Spain; Data Science for Health Services and Policy Research, Health Science Institute in Aragon (IACS), Aragon, Spain; Data Science for Health Services and Policy Research, Health Science Institute in Aragon (IACS), Aragon, Spain; Data Science for Health Services and Policy Research, Health Science Institute in Aragon (IACS), Aragon, Spain; Sciensano, Brussels, Belgium; Sciensano, Brussels, Belgium; Belgian Cancer Registry, Brussels, Belgium; Belgian Cancer Registry, Brussels, Belgium; Belgian Cancer Registry, Brussels, Belgium; Population Data Science, Swansea University Medical School, Faculty of Medicine, Health, and Life Science, Swansea University (SU), Swansea, United Kingdom; Population Data Science, Swansea University Medical School, Faculty of Medicine, Health, and Life Science, Swansea University (SU), Swansea, United Kingdom; Center of Epidemiology Biostatistics and Medical Information Technology, Department of Biomedical Sciences and Public Health, Università Politecnica delle Marche Home, Marche, Italy; Center of Epidemiology Biostatistics and Medical Information Technology, Department of Biomedical Sciences and Public Health, Università Politecnica delle Marche Home, Marche, Italy; Center of Epidemiology Biostatistics and Medical Information Technology, Department of Biomedical Sciences and Public Health, Università Politecnica delle Marche Home, Marche, Italy; Department of Cardiovascular and Endocrine-Metabolic Diseases and Aging, Istituto Superiore di Sanità, Italy; Department of Research and Health Statistics, Centre for Disease Prevention and Control (CDPC), Riga, Latvia; Department of Epidemiology and Health Monitoring, Robert Koch Institute, Berlin, Germany; Data Science for Health Services and Policy Research, Health Science Institute in Aragon (IACS), Aragon, Spain

## Abstract

**Background:**

The indirect impact of the coronavirus disease 2019 pandemic on healthcare services was studied by assessing changes in the trend of the time to first treatment for women 18 or older who were diagnosed and treated for breast cancer between 2017 and 2021.

**Methods:**

An observational retrospective longitudinal study based on aggregated data from four European Union (EU) countries/regions investigating the time it took to receive breast cancer treatment. We compiled outputs from a federated analysis to detect structural breakpoints, confirming the empirical breakpoints by differences between the trends observed and forecasted after March 2020. Finally, we built several segmented regressions to explore the association of contextual factors with the observed changes in treatment delays.

**Results:**

We observed empirical structural breakpoints on the monthly median time to surgery trend in Aragon (ranging from 9.20 to 17.38 days), Marche (from 37.17 to 42.04 days) and Wales (from 28.67 to 35.08 days). On the contrary, no empirical structural breakpoints were observed in Belgium (ranging from 21.25 to 23.95 days) after the pandemic's beginning. Furthermore, we confirmed statistically significant differences between the observed trend and the forecasts for Aragon and Wales. Finally, we found the interaction between the region and the pandemic's start (before/after March 2020) significantly associated with the trend of delayed breast cancer treatment at the population level.

**Conclusions:**

Although they were not clinically relevant, only Aragon and Wales showed significant differences with expected delays after March 2020. However, experiences differed between countries/regions, pointing to structural factors other than the pandemic.

## Introduction

### Population health information research infrastructure project

This study is part of the Population Health Information Research Infrastructure (PHIRI—https://www.phiri.eu/). PHIRI focused on generating and facilitating research on the health and well-being of the population after the pandemic (https://www.healthinformationportal.eu/). Four use cases were developed as part of the project. This study shows the results of the use case on delayed treatment in breast cancer patients during the coronavirus disease 2019 (COVID-19) pandemic. Specifically, this use case aimed to study whether the time to first treatment increased for women diagnosed with breast cancer—as a proxy for healthcare assistance to other cancer patients—as an indirect effect of the pandemic on the health systems following the reorganization of the health care services after the first surge of COVID cases.

### Background

The COVID-19 pandemic, declared by the World Health Organization (WHO)^1^ in March 2020, led to the need to reorganize healthcare in many countries.^2^ Particularly in Europe, the surge of COVID-19 cases forced some countries to reorganize healthcare services to cope with the large number of acute cases of COVID-19. As a result, countries reorganized their health systems, prioritizing the use of certain healthcare services, for instance, cancelling or postponing non-urgent and elective care.^3^ This reorganization of healthcare focused on prioritizing non-elective procedures such as transplants, cardiovascular surgery and cancer surgery.

Although the number of articles studying the effects of COVID-19 on public healthcare systems is limited,^4^ several studies have been carried out to measure the direct impact of the pandemic. Some of them focusing on cancer care.^5–7^ These studies analysed the variation in cancer screening participation rates between 2019 and 2020, showing a large decline.

On the other hand, other studies have described the impact of COVID-19 on cancer care as a decrease in cancer diagnoses and subsequent treatment^4^^,^^8^^,^^9^ during the pandemic. For instance, studies show that in Spain, between March and June 2020, there was a sharp decrease in the average number of diagnostic procedures for cancer compared to 2019, with a decrease of 57.1% in cytology and 41.2% in biopsies. They also noticed a 14.3% decrease in the average number of cancer patients treated daily in hospitals.^4^ Furthermore, a study by Cancer Research UK showed that the pandemic affected cancer care in two out of three patients and treatment in one out of three patients.^10^^,^^11^

### Aim of the international comparative analysis

The main aim of this study was to elicit changes in the trend of the time to first treatment in women diagnosed with breast cancer as an indirect effect of the COVID-19 pandemic, while also comparing potential differences among the participating countries/regions depending on their context and healthcare response.

## Methodology

### Federated analyses approach and intermediate local outputs

We used a federated approach to conduct this research project within PHIRI. The PHIRI federated research infrastructure (PHIRI FRI)^12^ is a network with a coordination node that orchestrates the workflow and communication between various federated nodes (i.e. research institutions and organizations) with access to sensitive health data. In this FRI, the coordination hub implemented and containerized the analysis pipeline in a standalone application deployed in each node. The nodes run the pipeline objects on the data sets prepared for the project and return the results to the orchestrating node for compilation and meta-analysis.

The workflow starts with materializing the research question in a data schema (i.e. information requirements) that becomes a common data model after some discussion rounds between the participating nodes. Upon agreement on the common data model, scripts for data quality assessment and algorithms for analysis are implemented and supported on a synthetic data set prepared following the specifications of the data model. Finally, all the digital objects in the pipeline (i.e. common data model, synthetic data set, data quality scripts and statistical algorithms) are containerized using Docker^13^ and deployed in the different nodes’ premises, where researchers run the analyses and devolve their results (local outputs) in the form of aggregated data. The common data model specification supporting this study^14^ and the analyses pipeline^15^ are publicly available at Zenodo.

### Study design

We used an observational retrospective longitudinal study based on aggregated data (local outputs from participant countries/regions analyses) on the distribution of the time interval between a breast cancer diagnosis in adult women (18 years old or older) to their first treatment (i.e. surgery, radiotherapy, chemotherapy, hormonotherapy and immunotherapy) at the population level in four EU countries/regions. We focused statistical analyses on surgery as it represented over 80% of the first treatment options in all regions/countries, incurring less variation over the study period. We conducted this study within the PHIRI. The EU countries/regions participating in this study were Aragon (AR, Spain), Belgium (BE, Belgium), Marche (MA, Italy), Wales (WA, UK), and Latvia (LV, Latvia). However, analyses and results from Latvia were excluded from this study after acknowledging data quality issues (see Supplementary material).

We compiled the aggregated outputs from the local analysis of the monthly distributions for time to first treatment in the participant countries and conducted the analyses considering (i) the monthly number of women treated, (ii) the monthly median and interquartile range of time to first treatment, distinguishing three different time series (surgery, radiotherapy or chemotherapy as first treatment) and (iii) descriptive characteristics of the women included in the study such as median, standard deviation and interquartile age range and the proportion of women with a low socioeconomic level.

### Statistical analyses

First, we calculated the directly standardized rates of treatment (*using the EU 27-countries standard population from Eurostat*^16^*as standard population*) by month, both globally and by type of treatment, to enable comparison among the participating regions. Secondly, we analysed the deseasonalized trends of median time-to-first-treatment to check for empirical structural breakpoints (BP) for each region/country. Then, we contrasted empirical breakpoints with the expected change point potentially produced by the surge of COVID-19 cases in March 2020.

Once we identified the empirical breakpoints, we forecasted the time series, using March 2020 as the expected inflexion point (considering the WHO declared the COVID-19 pandemic on 11 March 2020). We used the data before that date as a training set and after as a validation set for the prediction to assess the differences between the observed delays and the forecast. Again, we tested for statistically significant differences from the observed data to provide insight into each region’s direction and magnitude of the trend change. Finally, we built several segmented regression models considering potential contextual factors that could explain the observed changes in time-to-first-treatment trends. We checked for contextual factors such as the epidemiological data^17^ (i.e. monthly standardized cases), population health measures introduced as a response to the pandemic (i.e. monthly mode of the Oxford Stringency Index^18^), health system capacity (i.e. monthly median hospital admissions) and segmenting by period (i.e. before and after March 2020). We referenced the insights provided by these models to the empty model estimating median time-to-surgery. Finally, we conducted several sensitivity analyses considering the monthly standard rate of hospital and intensive care unit (ICU) admissions, other epidemiological data, and Google™ mobility trends.^19^ We compared them to select the model with the best performance.

To compare these time series across countries/regions, we needed to calculate the direct standardized rates. As the aggregate data output from the local analysis included only the median age and the number of treated women by period, we needed to reproduce a population from those parameters. To do this, we simulated the population of women with breast cancer using a normal distribution with median age as the mean and the interquartile range as the variance for the number of women treated per period (month), thus reconstructing the treated population by age group. Then, we calculated the direct standard rate using the region population and the European 27-countries standard population for each year (without the UK) during the study period.

### Structural breakpoint analysis in time series

Using the median time to first treatment (surgery) each month between 2017 and 2021 as a time series, we calculated the empirical breakpoints for both autocorrelation and periodicity of the series. Upon observing a strong seasonal component in the time series, we opted to decompose the time series and work only with the trend to conduct the structural breakpoints analysis. We aimed to identify empirically structural breakpoints after January 2020 that might be associated with the start of the COVID-19 pandemic in Europe. We estimated the BP on the time series trend, testing several models and selecting the model with the lowest Bayesian Information Criterion (BIC).

### Forecast after March 2020

We performed a forecast using data from the first through the 39th period (main break—March 2020) as the training set and the remainder periods as the validation set. The rationale behind this forecasting was to estimate whether the observed median times to surgery after March 2020 were within the 95% confidence interval of the forecast within each country. We tested several forecasting models until landing on a neural network time series forecast.

### Segmented regression analysis (interrupted time series)

Finally, we studied contextual factors associated with the observed trends. To do so, we built segmented regressions using a generalized linear mixed model with an instrument (i) whose value was ‘0’ before March 2020 and ‘1’ afterwards, signalling the start of the COVID-19 pandemic (i.e. segmentation). We estimated the median time to the first treatment (surgery), introducing contextual factors as independent variables for each region. We included the period, the median age of the women, the regional treatment standardized rate for surgery, the stringency index, the number of reported COVID-19 cases, the number of hospital and ICU admissions due to COVID-19 and mobility variation by location (i.e. retail and recreation, grocery shop and pharmacy, parks, station transit, work and residential areas) as potentially relevant contextual factors. Additionally, we reproduced a similar model considering only the region and the period as covariates, later introducing a random slope dependent on the segment (i), and an interaction between the country/region and segment (country | i), assessing model performance to gain insight on the main factors influencing the observed differences between countries/regions. Finally, we estimated the time to first treatment surgery for each region reproducing the initial model.

All the analyses were performed using R (*version 4.2.2*)^20^ within the RStudio (version *2022.07.1 + 554 Spotted Wakerobin—*more information in Supplementary materials).

## Results

### Descriptive analysis

**Figure 1:**
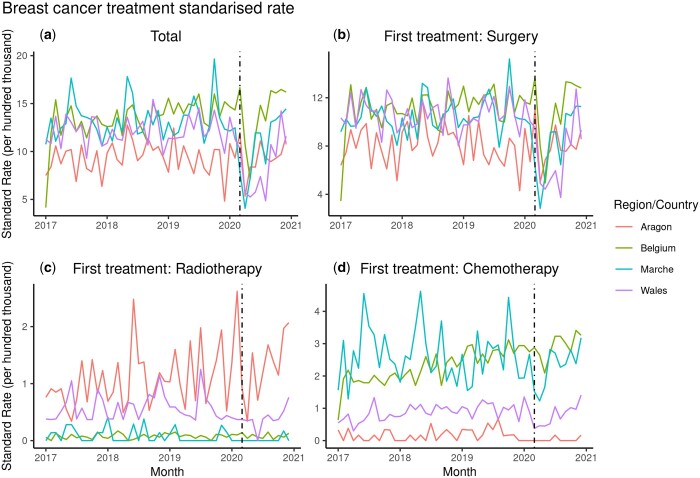
Directly standardized rates of treatment per hundred thousand women with breast cancer by type of treatment (a) total, (b) surgery, (c) radiotherapy and (d) chemotherapy in four EU countries/regions from January 2017 to December 2021. The vertical dotted line in March 2020 marks the start of the pandemic and the surge of COVID-19 cases in Europe

In the international comparison, the age distribution of women diagnosed with breast cancer who underwent treatment during the study period overlaps among the participant countries/regions, although showing some heterogeneity over time (Supplementary figure S1). Surgery was the first treatment for these patients in approximately 78%–84% of the cases in each country/region. Therefore, we focused on surgery as the first treatment for the subsequent statistical analyses (Supplementary figure S2). Direct standardized rates per hundred thousand women treated are compared among the participating countries/regions along the study period in figure 1.

**Figure 2:**
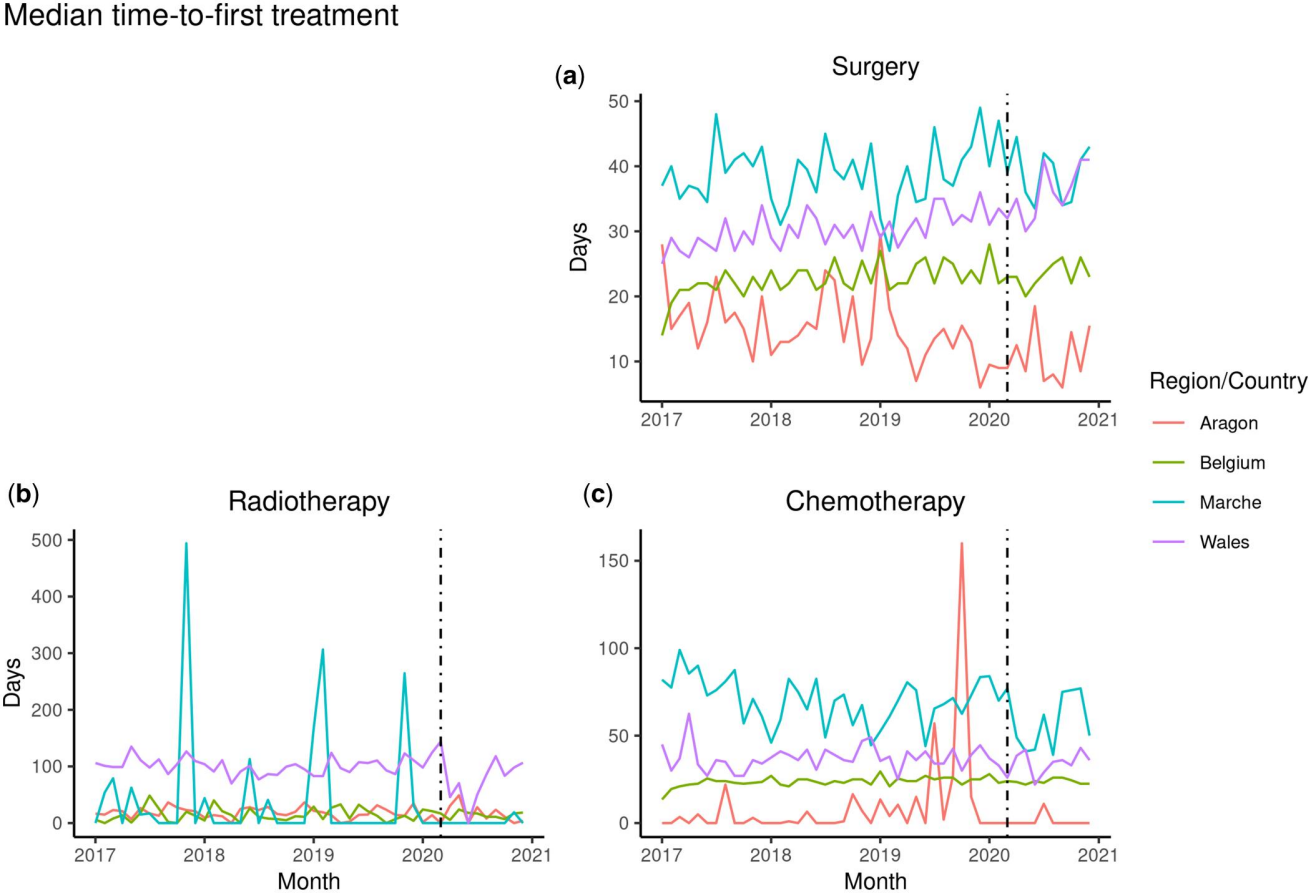
Monthly trend of median time from diagnosis to first treatment in women with breast cancer by type of treatment (a) surgery, (b) radiotherapy and (c) chemotherapy in four EU countries/regions from January 2017 to December 2021. The vertical dotted line in March 2020 marks the start of the pandemic and the surge of COVID-19 cases in Europe

We can observe a sharp decline in both the total treatments and in surgical procedure as the first treatment after March 2020 (dashed line) corresponding with the start of the pandemic (i.e. followed by lockdowns in several EU countries), and quickly recovering after June 2020. The differences in the trends of median time to first treatment (surgery) across countries/regions, observed in figure 2, may be due to how the time intervals were estimated for each country/region. However, independently of their data availability, the time to first treatment is measured consistently within each participant along the time series, thus enabling comparison and inference on potential events affecting the time series. In addition, these differences in scale among the participating countries/regions disappear when we do not consider the median times but the interquartile range of the distribution of times for each month along the period of study, as for surgery (Supplementary figure S3).

### Results of the structural breakpoints analyses

**Figure 3:**
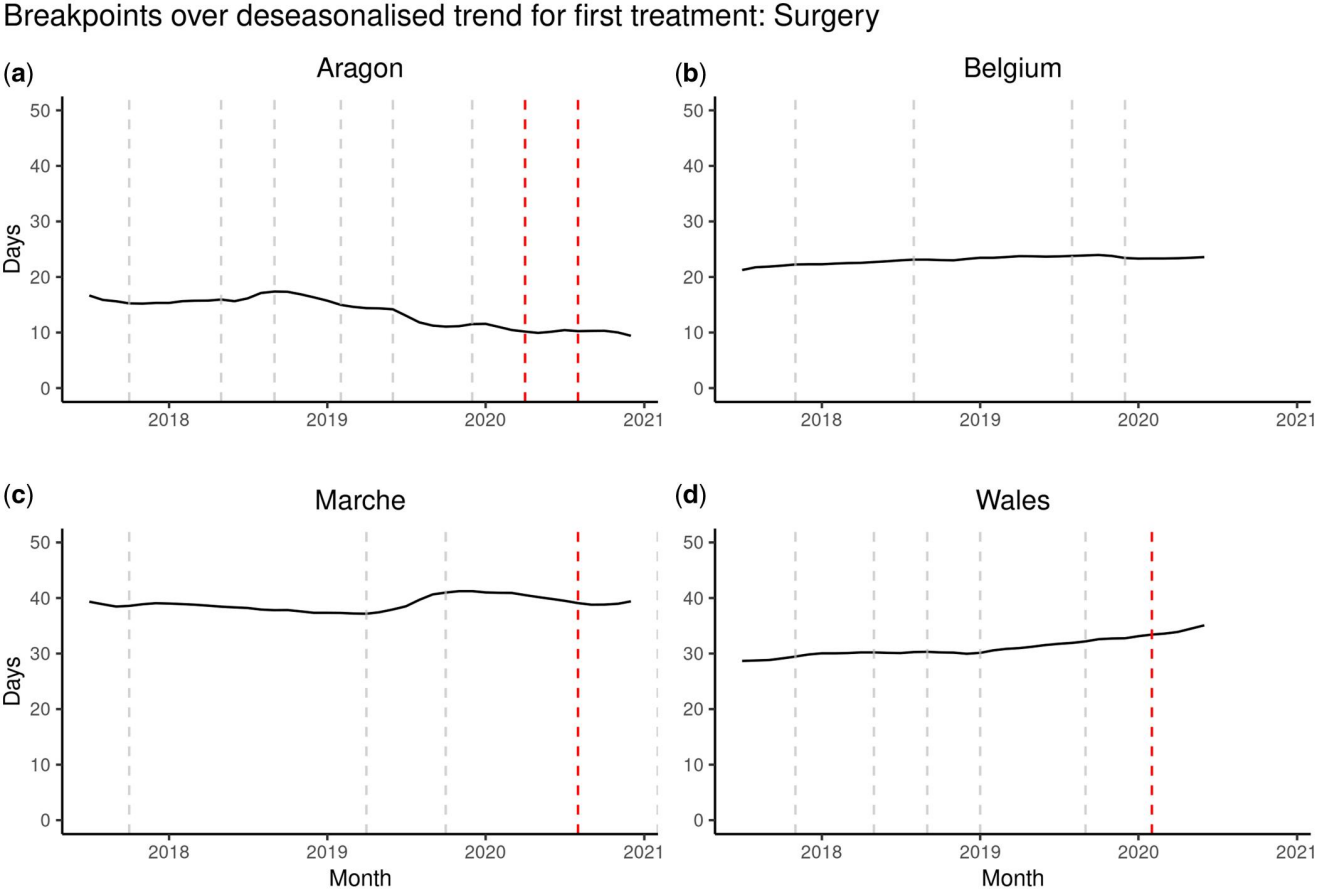
Breakpoints over deseasonalized monthly trend of median time-to-first-treatment: Surgery (in days) from January 2017 to December 2021 in (a) Aragon (Spain), (b) Belgium, (c) Marche (Italy) and (d) Wales (UK). Empirically detected structural breakpoints after January 2020 are highlighted in red to signal changes in the trend potentially compatible with an indirect impact of the COVID-19 pandemic on healthcare services

Considering the distribution of the delay to treatment in each country/region summarized by their median time to surgery by month (period), we can visualize a trend and analyse it as a time series in which we can identify certain empirical structural breakpoints, with special interest to those between January and December 2020 (marked by red dashed lines in figure 3). As shown in figure 3, we could identify breakpoints after January 2020 only in the regions of Aragon, Marche and the country of Wales. Focusing on those three countries/regions, the two breakpoints in March 2020 and July 2020 in Aragon might be associated with the pandemic's start and the posterior reorganization of health systems. However, the change in the median times was minimal (*less than one day (−0.52) from March 2020 to the end of the series*). In Marche, only one breakpoint appeared in August 2020, but again, the change in the median time is small (0.75 days from March 2020 to the end of the series). Finally, in Wales, only one breakpoint appears in January 2020, representing a slight change in the median time to the first treatment (1.5 days from March 2020 to the end of the series). However, none of the empirical breakpoints estimated were statistically significant when assessed (Supplementary figure S4).

As shown, variations in the median times (in days) were slight along the study period in Aragon (9.20, 17.38), Belgium (21.25, 23.95), Marche (37.17, 42.04) and Wales (28.67, 35.08).

Aragon and Wales showed the highest range in the median time to treatment values compared to Belgium and Marche, where the trend varied less, with the trend of Belgium and Wales slightly increasing while decreasing in Aragon.

### Results of the forecast

**Figure 4:**
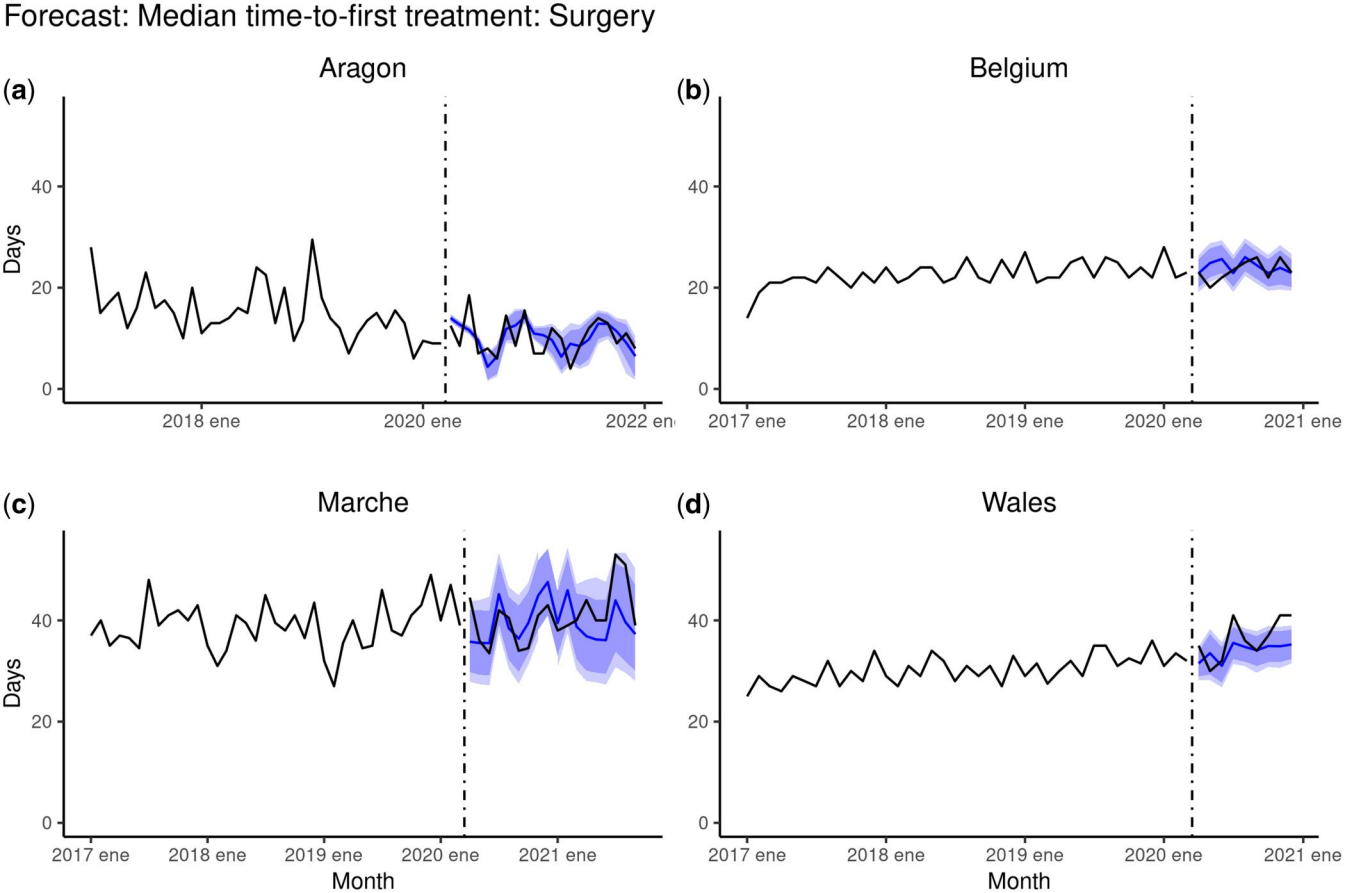
Forecast of the monthly trend of median time-to-first-treatment: Surgery (in days) from March 2020 onwards in (a) Aragon (Spain), (b) Belgium, (c) Marche (Italy) and (d) Wales (UK). The forecast was plotted as a solid blue line with 95 and 99% CI as shaded in dark blue and light blue areas, respectively. The vertical dotted line in March 2020 marks the start of the pandemic and the surge of COVID-19 cases in Europe

The results obtained for the forecast models are shown in figure 4.

We compared the observed trends with the forecasted trends based on the pre-pandemic data. We found statistically significant differences only in Aragon and Wales, indicating a potential impact of the COVID-19 pandemic on healthcare service performance (figure 4). On the other hand, observed trends in Belgium and Marche were within the forecast's confidence intervals (CI95% in dark blue and CI99% in light blue).

### Results of the segmented regression analyses

The outputs of the segmented regression analysis testing the association of the observed trends with several contextual factors are included in the regression models section as Supplementary materials. Most contextual factors were not significantly associated with the trend in the different models. On the one hand, when using Aragon as the reference (intercept), all countries/regions are statistically significant in predicting the median time to surgery for women with breast cancer. On the other hand, when looking at the output of the random effects, we observed a statistically significant association between the segment configured before and after March 2020, with another statistically significant association with the interaction between before and after March 2020 and the region. While in Aragon, the median time to treatment decreased after March 2020, in the other countries/regions, the median times increased slightly.

Finally, a summary of the performance of the models comparing all countries/regions shows that the model, including the interaction of before and after March 2020 and the region, had the best performance with a pseudo-*R*^2^ of 0.846 (see a comparison between models in Supplementary material).

## Discussion

In this study, we observed statistically significant changes in the time to first treatment trend in Aragon, Marche and Wales, showing several structural breakpoints after January 2020. However, these were not considered clinically relevant due to the short shift in the distribution (only a few days change in the median time to treatment). Despite this, other differences in trends were observed, including median times increasing in Belgium, Marche and Wales while decreasing in Aragon after the surge of COVID cases. Furthermore, the differences between the observed and the forecasted delays after March 2020 provided additional insight into the possible impact of the COVID-19 pandemic on healthcare. Finally, results from the segmented regression models showed that the period (month) variable turned statistically not significant when modelled along the region and accounting for the interaction between the region and the period before and after March 2020, which suggested that overall changes in the evolution of median time to surgery were more likely associated to existing structural factors at country/regional level.

The main factors associated with the pandemic's potential impact in healthcare, particularly in elective surgery, were identified as derived from the burden of hospitals and ICUs with COVID patients and the derivation of human and care resources to emergency assistance and support of COVID patients.^21^ Therefore, strict infection control procedures, limited hospital resources and infrastructures, prevalent physician and nurse shortages and increased demand led most countries and regions to implement guidelines and recommendations prioritizing surgery and overall specialized care treatments for certain severe conditions.^22^ The implementation of such Health System policies early in the pandemic drove healthcare reorganizations in each of the countries/regions participating in this study. For instance, in Spain, recommendations on safe surgery scheduling were issued by the Ministry of Health^23^ in June 2020 (updated in May 2021), prioritizing surgery in cancer patients. Similar recommendations were also issued at difference instances of their respective Health Systems in Wales, Belgium and Marche (Italy),^24–26^ not without criticisms about their implementation in some cases.^27^

The COVID-19 pandemic has posed unprecedented challenges for healthcare management of breast cancer patients, affecting their access to timely and appropriate diagnosis and treatment. However, most studies measuring the impact of the pandemic on this population have focused on the potential causes or factors for disruption, the delay in the diagnosis of new cases by the disruption of the population screening programs, or the potential outcomes of the delay, such as increased severity, morbidity and complexity of the cases potentially leading to increased mortality. Few studies have specifically addressed the delay to treat those breast cancer patients who were already diagnosed before or during the pandemic.^28–31^ Observed results are compatible with those published in the literature but are non-comparable based on structural differences on the healthcare systems between participant countries (in Europe) and studies conducted in the USA.

### Strengths and limitations

Strengths of this study include the large cohort covering all women with breast cancer treated in four EU countries/regions between 2017 and 2020 (during at least 48 months), producing comparable standardized rates of treatment for each country/region, the inclusion of a range of contextual factors potentially associated with the trend of treatment delay considering both healthcare offer (i.e. case burden due to COVID-19 cases, admissions to hospital or ICU), and healthcare demand factors (i.e. stringency index, mobility, etc.); and the novelty of the federated approach facilitating comparative analysis of routinely collected health data from diverse sources.

A first limitation is that we used aggregated data summarizing the distribution of treated women, thus disregarding other indirect effects of the COVID-19 pandemic in terms of diagnosis delay or decline in the number of diagnoses over time.^32^ We also relied on the monthly median to characterize the trend along the study period of treatment delay, therefore limiting our capacity to study the evolution of variations over time.

A third limitation of this study is that we decided to exclude data from Latvia. We considered quality issues regarding comparability due to a lack of access to data sources other than hospital episodes challenging the identification of the original date of the breast cancer diagnosis and the correct identification of the first treatment to calculate the interval as the time to first treatment. Organizational interoperability, which involves coordination in health data stewardship between different institutions, was a major factor in accessing and linking relevant data to achieve these results. Although each country/region used, the same definition and the interquartile range of time to surgery overlapped for all countries/regions (see Supplementary figure S2), some variation in the calculation of this delay can be attributed to the availability of data and the correct identification of both diagnosis and intervention.

A final limitation is that this study was initially planned as a feasibility study to test the federated approach to conducting population health research to inform European health policy. Thus, we focused on assessing the potential indirect impact of the pandemic however; we did not follow up analysing the actual guidelines reorganizing healthcare service assistance to prioritize certain conditions in the context of pressed or scarce resources. Further research is needed on the specific healthcare policies implemented in each region/country.

### Implications

Our study shows that statistically significant changes in trends of the time to first treatment for women with breast cancer during the pandemic were only observed in two of the four countries/regions analysed, which showed different behaviours, and were associated with none of the other contextual factors, tested at the regional level, besides the response of the region to the surge of COVID-19 cases.

Finally, this study highlights the feasibility of using a federated approach to analyse routinely collected real-world health data to produce valuable scientific evidence informing health policy and supporting preparedness for future health crises.

## Supplementary Material

ckae038_Supplementary_Data

## Data Availability

The data underlying this article are available in ZENODO, at https://doi.org/10.5281/zenodo.6724454

## Disclaimer

These results correspond to the international comparison analysis for countries participating in PHIRI WP6 Use Case B on delayed treatment of women with breast cancer as an indirect effect of the COVID-19 pandemic. A demonstrator of the analytical pipeline implemented for Use Case B is available as a Demonstrator in the Health Information Portal complemented with additional information (https://www.healthinformationportal.eu/services/federated-demonstrators/phiri-use-case-b-demonstrator). The use case B report is updated iteratively and automatically each time a new country completes the local analysis of their data using the PHIRI app (https://doi.org/10.5281/zenodo.5729310), upon uploading the aggregated outputs using the on-line interface available at https://phiri.iacs.es/upload. The content of this article represents the views of the authors only and is their sole responsibility. The European Research Executive Agency (REA) and the European Commission are not responsible for any use that may be made of the information it contains.

Key points

- Structural breakpoints, although not statistically significant, can be empirically observed in the trend of treatment delay for breast cancer after January 2020 in several EU countries/regions.
- We can observe statistically significant changes in the trend of treatment delay immediately after the beginning of the COVID-19 pandemic for Aragon and Wales, although they are not clinically relevant.
- These changes do not seem to be associated with several contextual factors at the population level, such as mobility, health policy measures stringency index, or epidemiological data.
- The present study demonstrates that a federated approach is feasible for analysing routinely collected real-world health data to produce valuable evidence informing health policy.
