# Supplementary material for: Delayed treatment in breast cancer patients during the COVID-19 pandemic: a population health information research infrastructure (PHIRI) case study
Source: Eur J Public Health. 2024 Jul 1;34(Suppl 1):i50–7. doi: 10.1093/eurpub/ckae038 (PMC11215325; doi:10.1093/eurpub/ckae038)
Supplement: ckae038_Supplementary_Data [file ckae038_supplementary_data.zip › ejph-2023-06-phis-0276-File006.docx]

## Supplementary materials

|  | 2017 | 2018 | 2019 | 2020 | 2021 | Total |
| --- | --- | --- | --- | --- | --- | --- |
| Belgium | 6980 | 7569 | 8320 | 8046 | 0* | 30915 |
| Aragón | 600 | 708 | 620 | 671 | 750 | 3349 |
| Wales | 1967 | 1960 | 1978 | 1475 | 0* | 7380 |
| Marche | 1123 | 1173 | 1191 | 956 | 768****** | 5211 |
| Latvia | 1932 | 1573 | 1560 | 35 | 4* | 5104 |

*Table S1: Total number of women with breast cancer treated included in the cohort for the analysis at the population level (cohort sample size) by region and year during the study period.*

** Data from Belgium, Wales and Latvia in 2021 were not available for analysis at the time of producing the analysis for this study.*

***Data from Marche in 2021 includes only women with breast cancer treated in the region from January to September.*


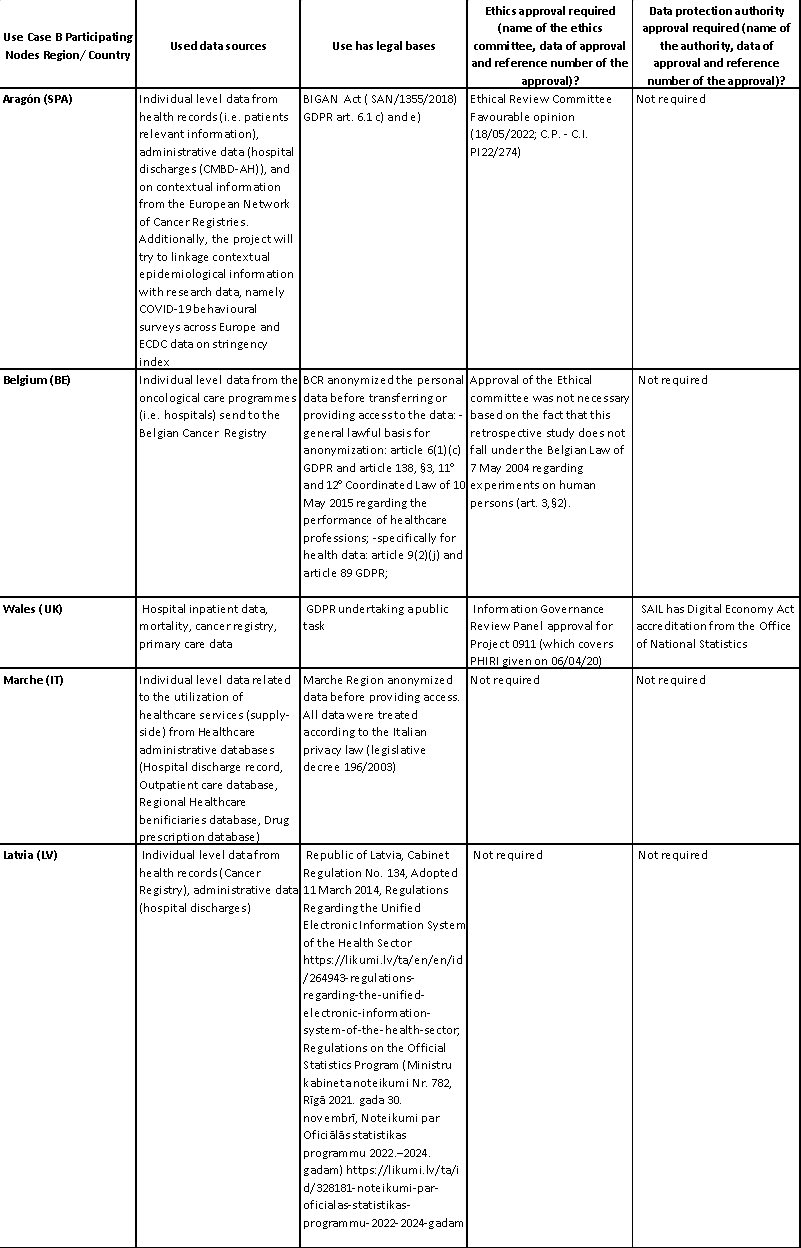


*Table S2: Summary of data sources, legal bases for using original data, requirement for ethical approval and requirement for data protection authority approval by participating region/country.*


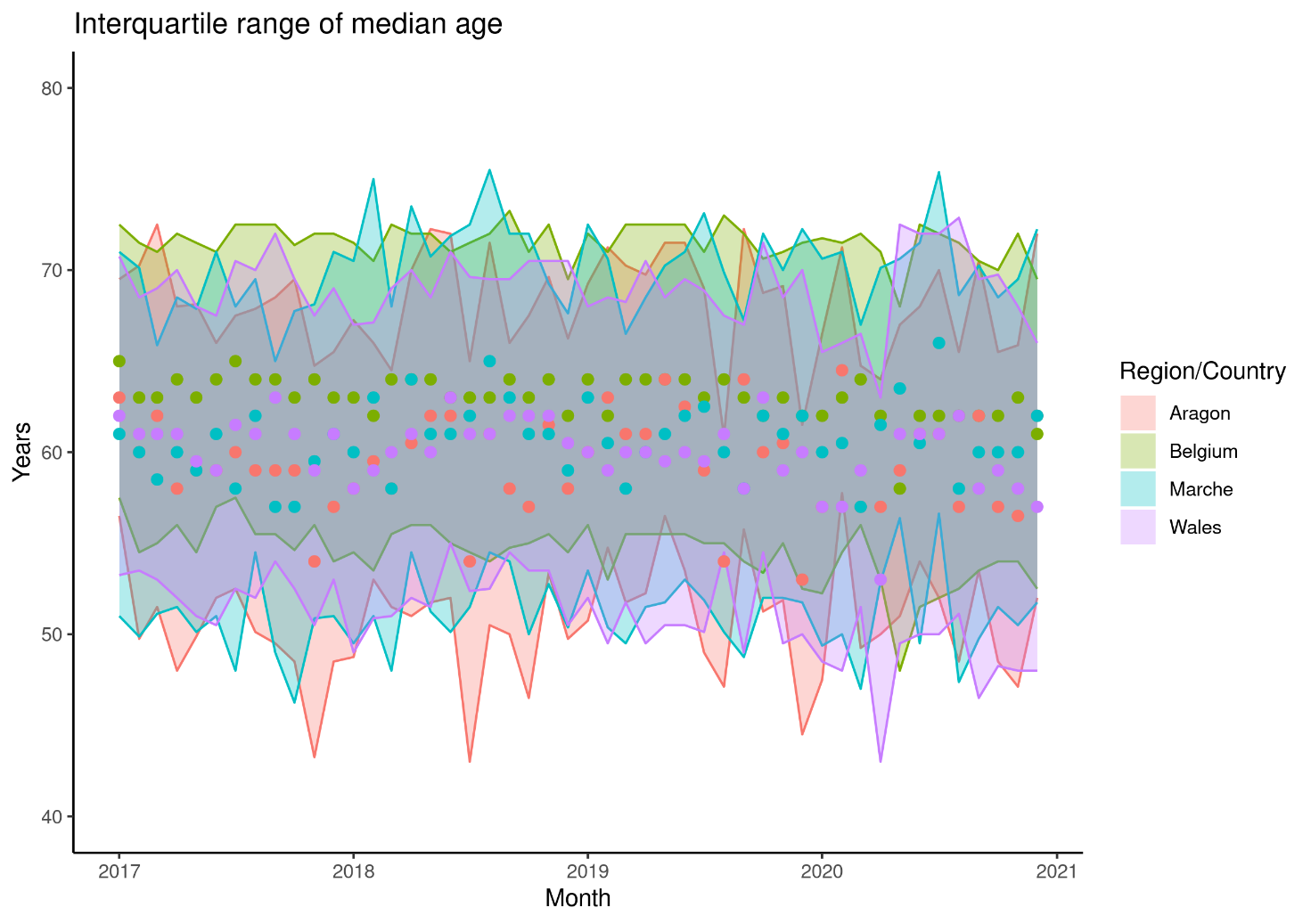


*Figure S1: Interquartile range of monthly median ages by region during the study period*


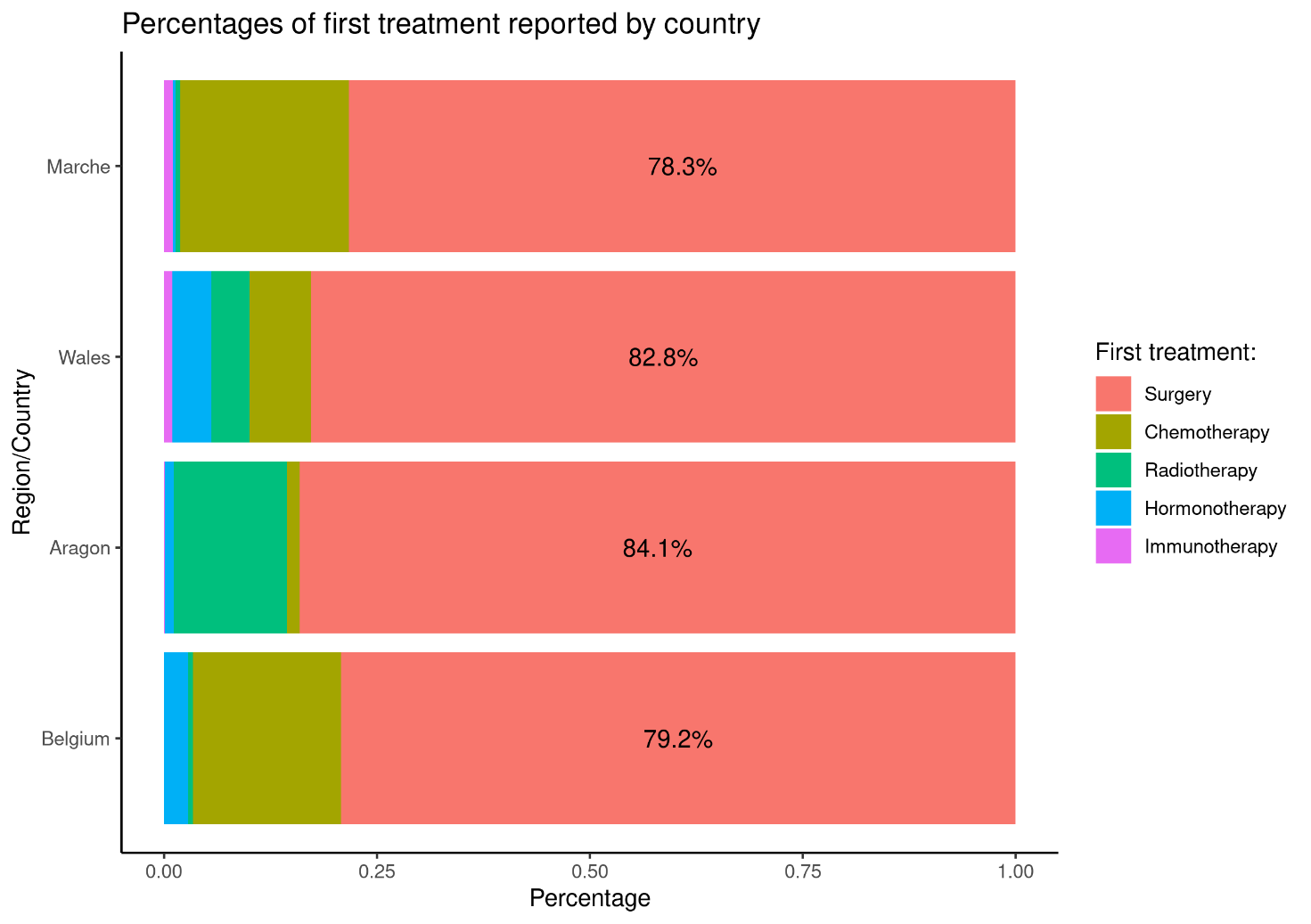


*Figure S2: Percentage of women with breast cancer receiving each type of treatment as first treatment by region during the study period*


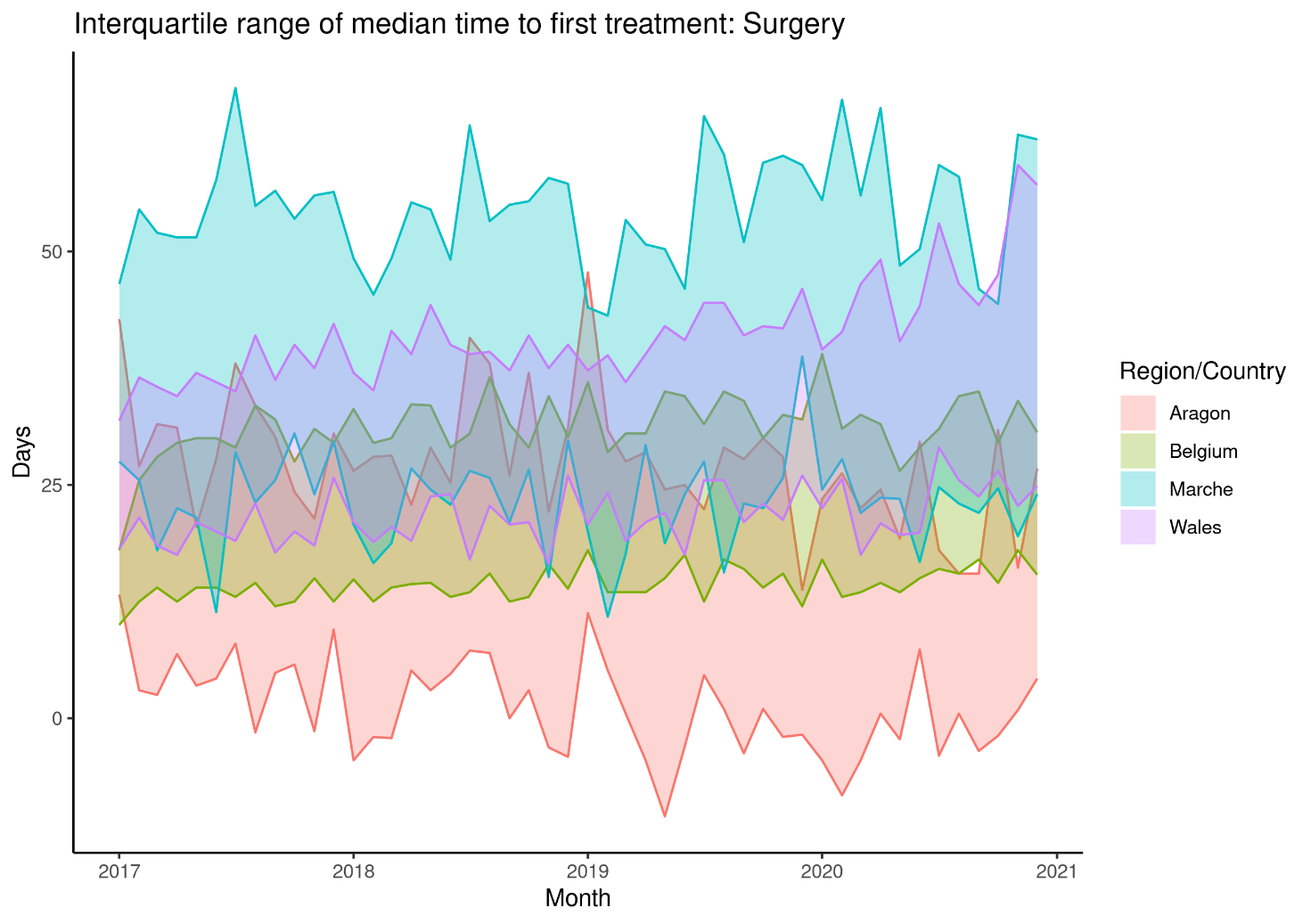


*Figure S3: Interquartile range of time to first treatment surgery in women with breast cancer by region during the study period*


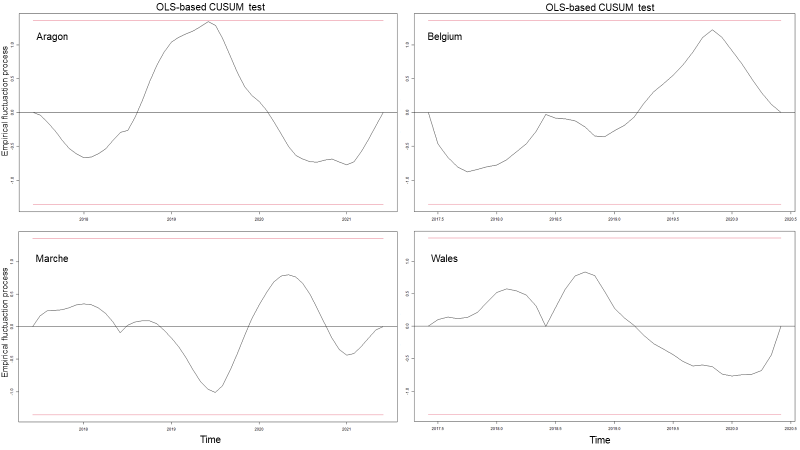


*Figure S4: OLS-based CUSUM test of the structural breakpoint analysis over the deseasonalised monthly trend of median time-to-first-treatment: Surgery (in days) in Aragon (Spain), Belgium, Marche (Italy), and Wales.*


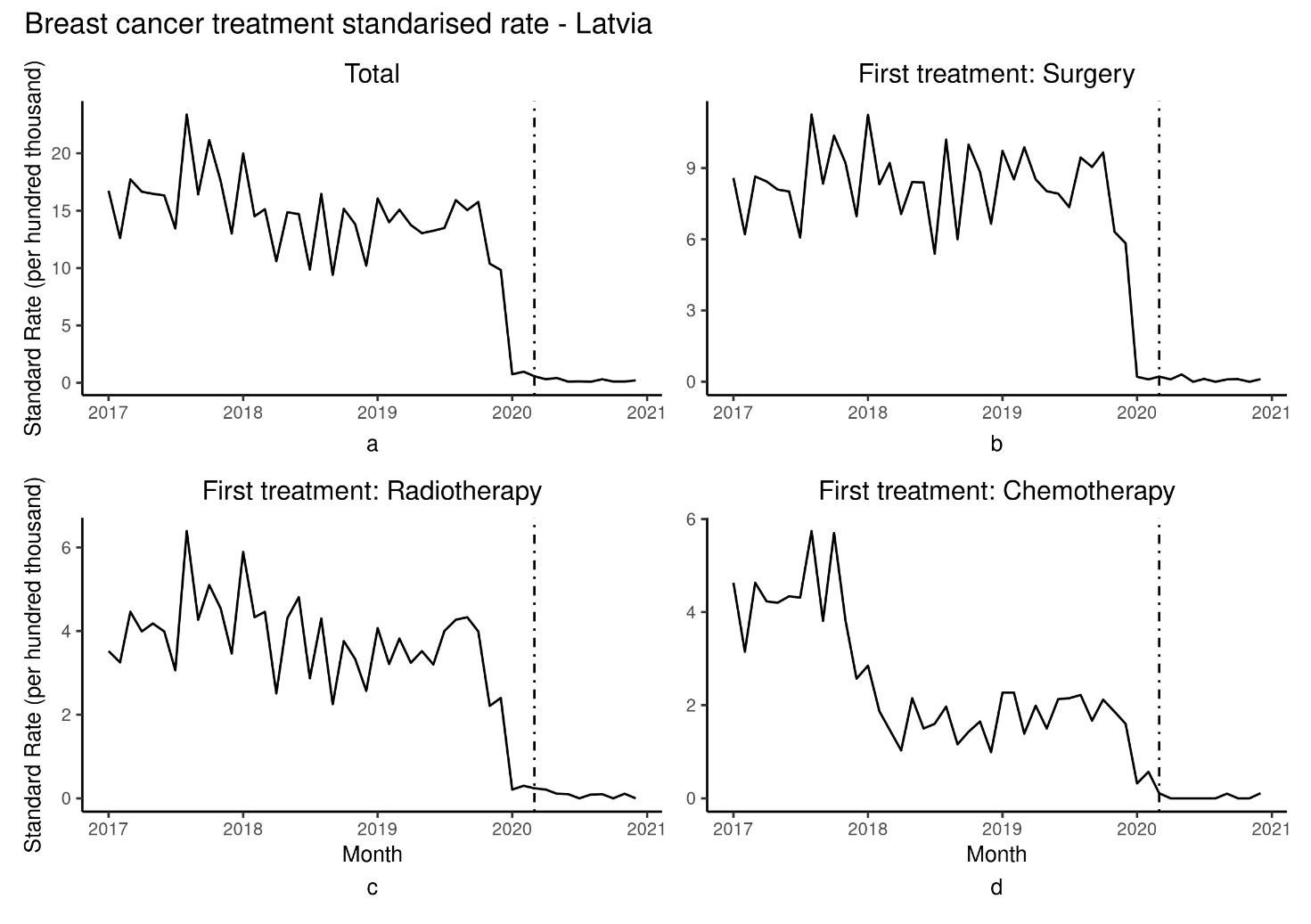


*Figure supp Latvia 1: Breast cancer treatment standardised rates for Latvia by type of treatment during the study period*
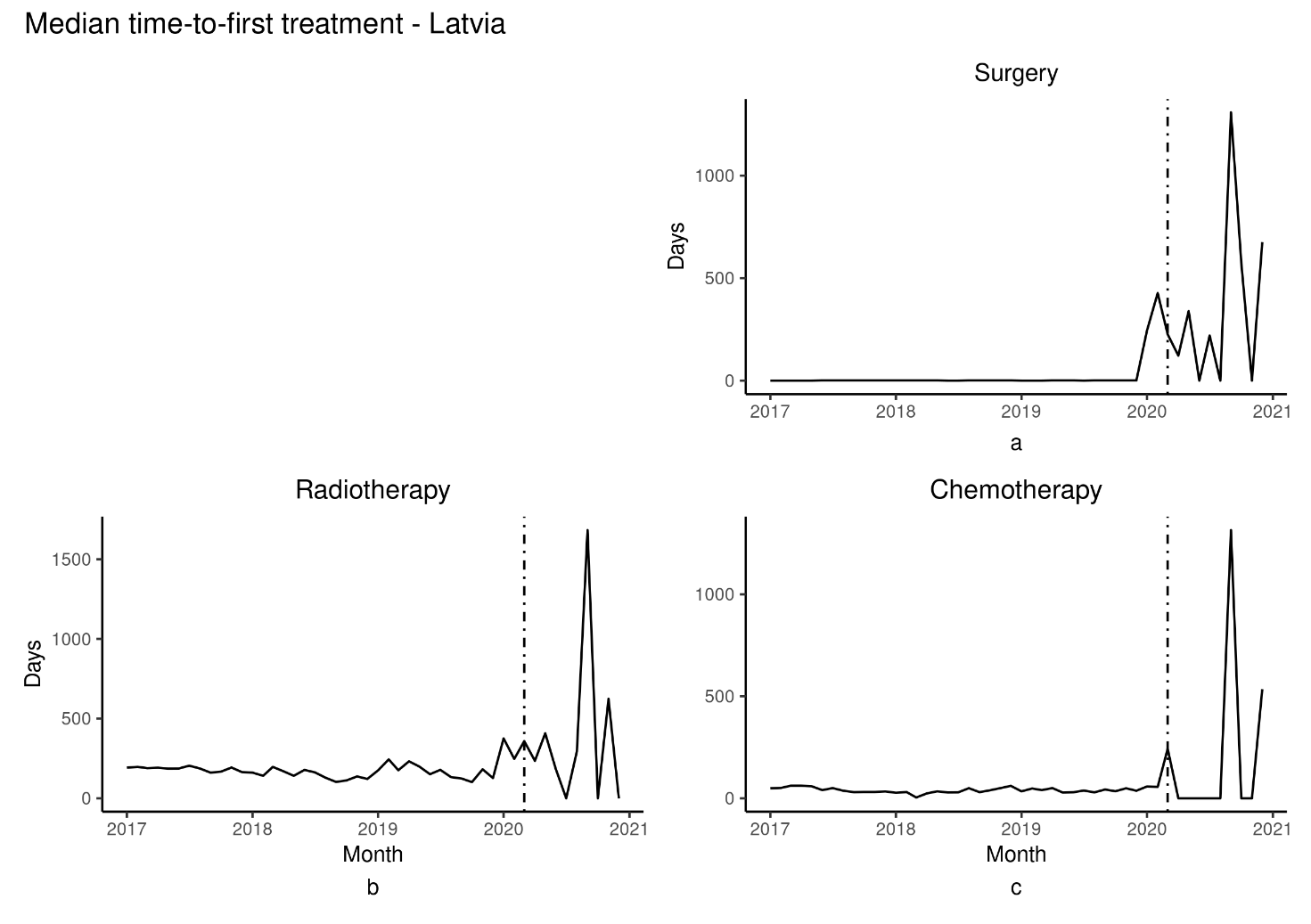


*Figure supp Latvia 2: Median time to first treatment for Latvia by type of treatment during the study period*

### Regression Models

Regression models with data for each country/region individually:

- Aragon:


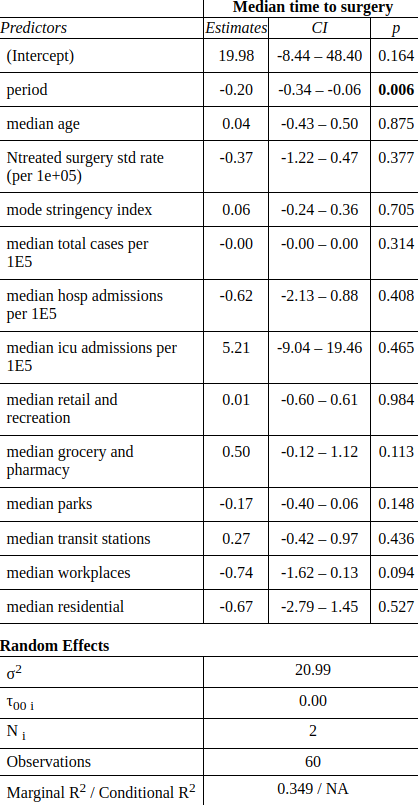


*Figure Supp Regression Models 1: Regression Model with all variables for Aragon*

- Belgium:


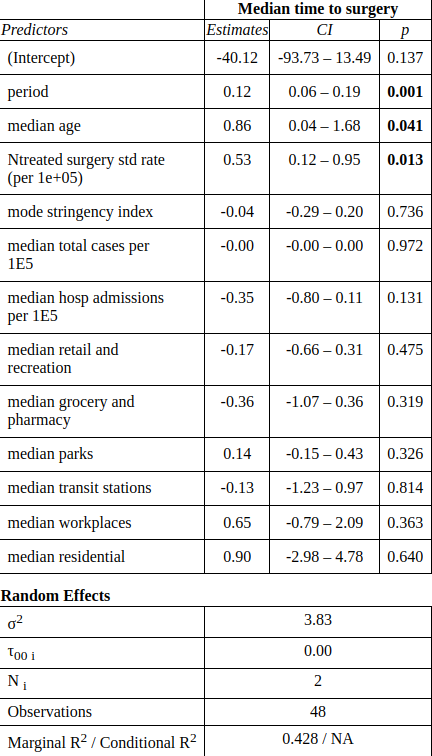


*Figure Supp Regression Models 2: Regression Model with all variables for Belgium*

- Marche:


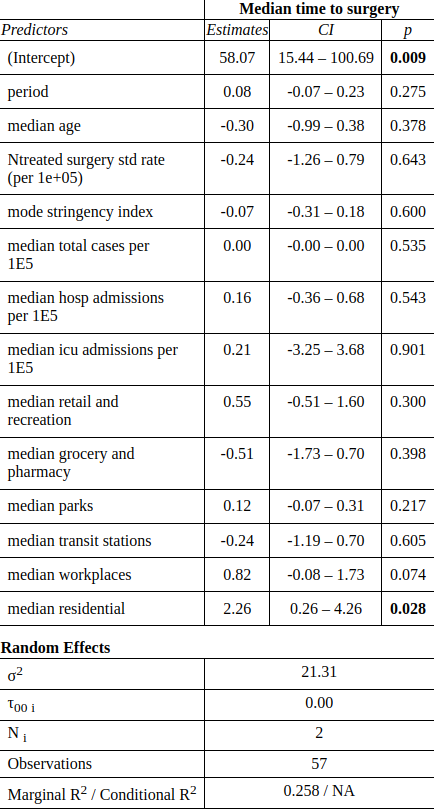


*Figure Supp Regression Models 3: Regression Model with all variables for Marche*

- Wales:


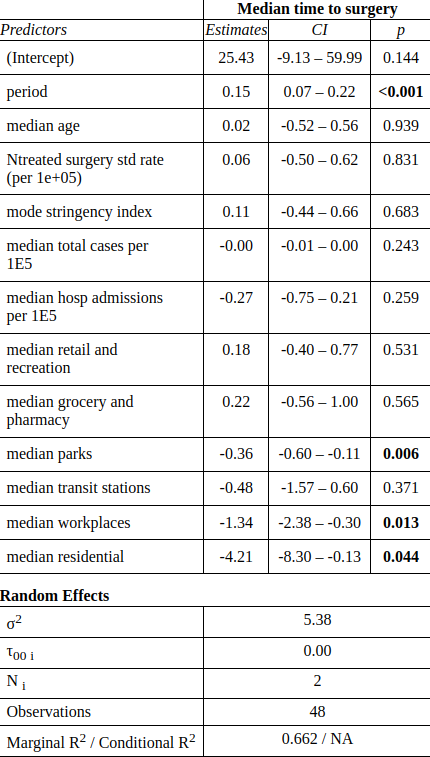


*Figure Supp Regression Models 4: Regression Model with all variables for Wales*

Regression models with all participant data, including region (country) as variable:

- Model 0: with all variables


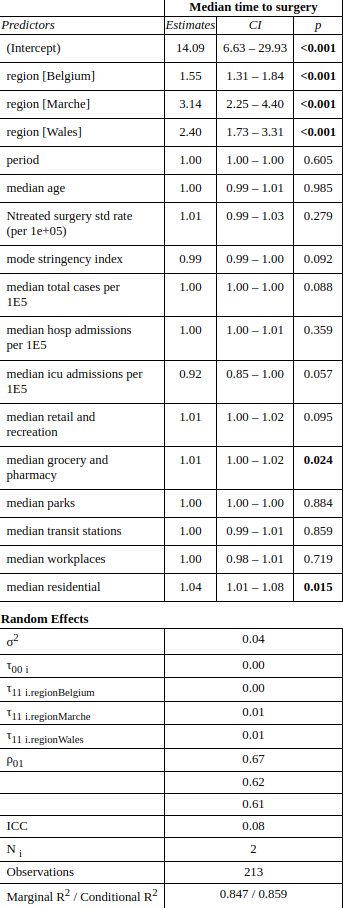


*Figure Supp Regression Models 5: Regression Model 0 with all variables*

- Model 1: with only region variable


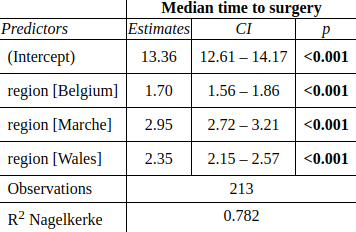


*Figure Supp Regression Models 6: Regression Model 1 with only region variable*

- Model 2: with region and interaction with region


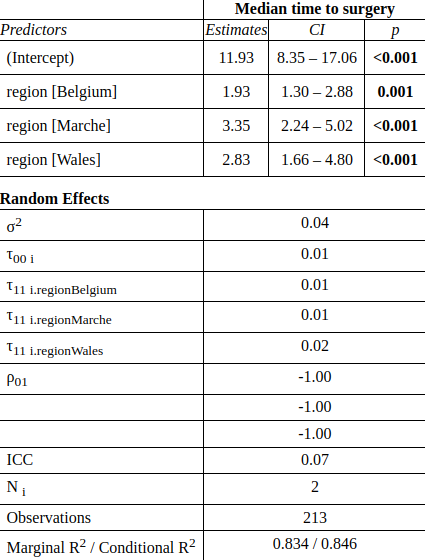


*Figure Supp Regression Models 7: Regression Model 2 with region and interaction with*

*region*

- Model 3: with region, period and interaction with region


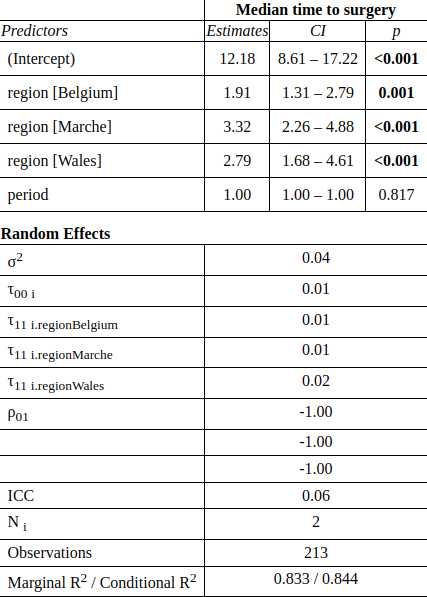


*Figure Supp Regression Models 8: Regression Model 3 with region, period and interaction with region*

- Model 4: with region, contextual factors and interaction with region


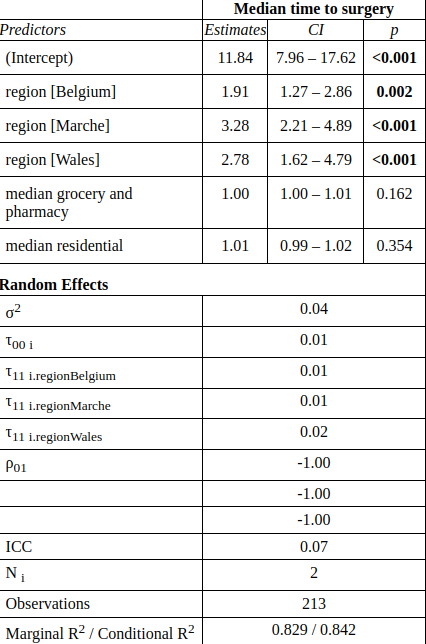


*Figure Supp Regression Models 9: Regression Model 4 with region, contextual factors and interaction with region*

- Model 5: with the region, period, contextual factors and interaction with the region


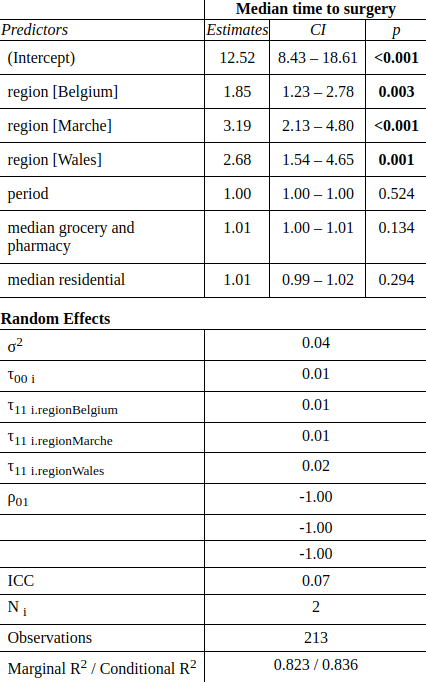


*Figure Supp Regression Models 10: Regression Model 5 with the region, period, contextual factors and interaction with the region*

- Comparison models: comparison between the 5 models

| **Name** | **Model** | **RMSE** | **Sigma** | **AIC weights** | **AICc weights** | **BIC weights** | **Performance-Score** |
| --- | --- | --- | --- | --- | --- | --- | --- |
| Model 2 | glmerMod | 3928 | 208 | 510 | 586 | 2 | 72.59 % |
| Model 4 | glmerMod | 3825 | 208 | 206 | 167 | 2.99e-05 | 51.31% |
| Model 3 | glmerMod | 3948 | 208 | 193 | 187 | 1.50e-04 | 45.64% |
| Model 5 | glmerMod | 3870 | 207 | 91 | 61 | 2.45e-06 | 41.74% |
| Model 0 | glmerMod | 4047 | 204 | 1.43e-04 | 6.71e-06 | 1.93e-16 | 30.36% |
| Model 1 | glm | 4286 | 231 | 1.20e-05 | 4.04e-05 | 998 | 20.00% |

*Table Supp Regression Models 1: Comparison between the 5 models ordered by performance*

We used R version 4.2.2 (R Core Team 2022) and the following R packages: changepoint v. 2.2.3 (Killick and Eckley 2014; Killick, Haynes, and Eckley 2022), effects v. 4.2.2 (Fox 2003; Fox and Hong 2009; Fox and Weisberg 2018, 2019), epitools v. 0.5.10.1 (Aragon 2020), eurostat v. 3.7.10 (Lahti et al. 2017), fable v. 0.3.2 (O’Hara-Wild, Hyndman, and Wang 2022a), feasts v. 0.3.0 (O’Hara-Wild, Hyndman, and Wang 2022b), forecast v. 8.17.0 (R. J. Hyndman and Khandakar 2008; R. Hyndman et al. 2022), ggeffects v. 1.1.3 (Lüdecke 2018a), gghighlight v. 0.3.3 (Yutani 2022), grateful v. 0.1.11 (Rodríguez-Sánchez, Jackson, and Hutchins 2022), gridExtra v. 2.3 (Auguie 2017), hglm v. 2.2.1 (Ronnegard, Shen, and Alam 2010; Alam, Ronnegard, and Shen 2015), Hmisc v. 4.7.1 (Harrell Jr 2022), jtools v. 2.2.1 (Long 2022), knitr v. 1.41 (Xie 2014, 2015, 2022), lme4 v. 1.1.31 (Bates et al. 2015), lmerTest v. 3.1.3 (Kuznetsova, Brockhoff, and Christensen 2017), mgcv v. 1.8.41 (S. N. Wood 2003, 2004, 2011; S. N. Wood et al. 2016; S. N. Wood 2017), modeest v. 2.4.0 (Poncet 2019), patchwork v. 1.1.2 (Pedersen 2022), performance v. 0.10.0 (Lüdecke, Ben-Shachar, et al. 2021), plotly v. 4.10.0 (Sievert 2020), prophet v. 1.0 (Taylor and Letham 2021), reshape2 v. 1.4.4 (Wickham 2007), rmarkdown v. 2.18 (Xie, Allaire, and Grolemund 2018; Xie, Dervieux, and Riederer 2020; Allaire et al. 2022), see v. 0.7.2 (Lüdecke, Patil, et al. 2021), sjlabelled v. 1.2.0 (Lüdecke 2022a), sjmisc v. 2.8.9 (Lüdecke 2018b), sjPlot v. 2.8.11 (Lüdecke 2022b), strucchange v. 1.5.3 (Zeileis et al. 2002, 2003; Zeileis 2006), styler v. 1.7.0 (Müller and Walthert 2022), tidyverse v. 1.3.2 (Wickham et al. 2019), tsfeatures v. 1.0.2 (R. Hyndman et al. 2020), tsibble v. 1.1.3 (Wang, Cook, and Hyndman 2020), zoo v. 1.8.11 (Zeileis and Grothendieck 2005).

## List of R packages and version used

| **Package** | **Version** | **Citation** |
| --- | --- | --- |
| base | 4.2.2 | R Core Team (2022) |
| changepoint | 2.2.3 | Killick and Eckley (2014); Killick, Haynes, and Eckley (2022) |
| effects | 4.2.2 | Fox (2003); Fox and Hong (2009); Fox and Weisberg (2018); Fox and Weisberg (2019) |
| epitools | 0.5.10.1 | Aragon (2020) |
| eurostat | 3.7.10 | Lahti et al. (2017) |
| fable | 0.3.2 | O’Hara-Wild, Hyndman, and Wang (2022a) |
| feasts | 0.3.0 | O’Hara-Wild, Hyndman, and Wang (2022b) |
| forecast | 8.17.0 | R. J. Hyndman and Khandakar (2008); R. Hyndman et al. (2022) |
| ggeffects | 1.1.3 | Lüdecke (2018a) |
| gghighlight | 0.3.3 | Yutani (2022) |
| grateful | 0.1.11 | Rodríguez-Sánchez, Jackson, and Hutchins (2022) |
| gridExtra | 2.3 | Auguie (2017) |
| hglm | 2.2.1 | Ronnegard, Shen, and Alam (2010); Alam, Ronnegard, and Shen (2015) |
| Hmisc | 4.7.1 | Harrell Jr (2022) |
| jtools | 2.2.1 | Long (2022) |
| knitr | 1.41 | Xie (2014); Xie (2015); Xie (2022) |
| lme4 | 1.1.31 | Bates et al. (2015) |
| lmerTest | 3.1.3 | Kuznetsova, Brockhoff, and Christensen (2017) |
| mgcv | 1.8.41 | S. N. Wood (2003); S. N. Wood (2004); S. N. Wood (2011); S. N. Wood et al. (2016); S. N. Wood (2017) |
| modeest | 2.4.0 | Poncet (2019) |
| patchwork | 1.1.2 | Pedersen (2022) |
| performance | 0.10.0 | Lüdecke, Ben-Shachar, et al. (2021) |
| plotly | 4.10.0 | Sievert (2020) |
| prophet | 1.0 | Taylor and Letham (2021) |
| reshape2 | 1.4.4 | Wickham (2007) |
| rmarkdown | 2.18 | Xie, Allaire, and Grolemund (2018); Xie, Dervieux, and Riederer (2020); Allaire et al. (2022) |
| see | 0.7.2 | Lüdecke, Patil, et al. (2021) |
| sjlabelled | 1.2.0 | Lüdecke (2022a) |
| sjmisc | 2.8.9 | Lüdecke (2018b) |
| sjPlot | 2.8.11 | Lüdecke (2022b) |
| strucchange | 1.5.3 | Zeileis et al. (2002); Zeileis et al. (2003); Zeileis (2006) |
| styler | 1.7.0 | Müller and Walthert (2022) |
| tidyverse | 1.3.2 | Wickham et al. (2019) |
| tsfeatures | 1.0.2 | R. Hyndman et al. (2020) |
| tsibble | 1.1.3 | Wang, Cook, and Hyndman (2020) |
| zoo | 1.8.11 | Zeileis and Grothendieck (2005) |

## Package citations

Alam, Moudud, Lars Ronnegard, and Xia Shen. 2015. “Fitting Conditional and Simultaneous Autoregressive Spatial Models in Hglm.” *The R Journal* 7 (2): 5–18. <https://journal.r-project.org/archive/2015/RJ-2015-017/RJ-2015-017.pdf>.

Allaire, JJ, Yihui Xie, Jonathan McPherson, Javier Luraschi, Kevin Ushey, Aron Atkins, Hadley Wickham, Joe Cheng, Winston Chang, and Richard Iannone. 2022. *Rmarkdown: Dynamic Documents for r*. <https://github.com/rstudio/rmarkdown>.

Aragon, Tomas J. 2020. *Epitools: Epidemiology Tools*. [https://CRAN.R-project.org/package=epitools](https://cran.r-project.org/package=epitools).

Auguie, Baptiste. 2017. *gridExtra: Miscellaneous Functions for "Grid" Graphics*. [https://CRAN.R-project.org/package=gridExtra](https://cran.r-project.org/package=gridExtra).

Bates, Douglas, Martin Mächler, Ben Bolker, and Steve Walker. 2015. “Fitting Linear Mixed-Effects Models Using lme4.” *Journal of Statistical Software* 67 (1): 1–48. <https://doi.org/10.18637/jss.v067.i01>.

Fox, John. 2003. “Effect Displays in R for Generalised Linear Models.” *Journal of Statistical Software* 8 (15): 1–27. <https://doi.org/10.18637/jss.v008.i15>.

Fox, John, and Jangman Hong. 2009. “Effect Displays in R for Multinomial and Proportional-Odds Logit Models: Extensions to the effects Package.” *Journal of Statistical Software* 32 (1): 1–24. <https://doi.org/10.18637/jss.v032.i01>.

Fox, John, and Sanford Weisberg. 2018. “Visualizing Fit and Lack of Fit in Complex Regression Models with Predictor Effect Plots and Partial Residuals.” *Journal of Statistical Software* 87 (9): 1–27. <https://doi.org/10.18637/jss.v087.i09>.

———. 2019. *An r Companion to Applied Regression*. 3rd ed. Thousand Oaks CA: Sage. <https://socialsciences.mcmaster.ca/jfox/Books/Companion/index.html>.

Harrell Jr, Frank E. 2022. *Hmisc: Harrell Miscellaneous*. [https://CRAN.R-project.org/package=Hmisc](https://cran.r-project.org/package=Hmisc).

Hyndman, Rob J, and Yeasmin Khandakar. 2008. “Automatic Time Series Forecasting: The Forecast Package for R.” *Journal of Statistical Software* 26 (3): 1–22. <https://doi.org/10.18637/jss.v027.i03>.

Hyndman, Rob, George Athanasopoulos, Christoph Bergmeir, Gabriel Caceres, Leanne Chhay, Mitchell O’Hara-Wild, Fotios Petropoulos, Slava Razbash, Earo Wang, and Farah Yasmeen. 2022. *forecast: Forecasting Functions for Time Series and Linear Models*. <https://pkg.robjhyndman.com/forecast/>.

Hyndman, Rob, Yanfei Kang, Pablo Montero-Manso, Thiyanga Talagala, Earo Wang, Yangzhuoran Yang, and Mitchell O’Hara-Wild. 2020. *Tsfeatures: Time Series Feature Extraction*. [https://CRAN.R-project.org/package=tsfeatures](https://cran.r-project.org/package=tsfeatures).

Killick, Rebecca, and Idris A. Eckley. 2014. “changepoint: An R Package for Changepoint Analysis.” *Journal of Statistical Software* 58 (3): 1–19. <https://www.jstatsoft.org/v58/i03/>.

Killick, Rebecca, Kaylea Haynes, and Idris A. Eckley. 2022. *changepoint: An R Package for Changepoint Analysis*. [https://CRAN.R-project.org/package=changepoint](https://cran.r-project.org/package=changepoint).

Kuznetsova, Alexandra, Per B. Brockhoff, and Rune H. B. Christensen. 2017. “lmerTest Package: Tests in Linear Mixed Effects Models.” *Journal of Statistical Software* 82 (13): 1–26. <https://doi.org/10.18637/jss.v082.i13>.

Lahti, Leo, Janne Huovari, Markus Kainu, and Przemyslaw Biecek. 2017. “Retrieval and Analysis of Eurostat Open Data with the Eurostat Package.” *The R Journal* 9 (1): 385–92. <https://doi.org/10.32614/RJ-2017-019>.

Long, Jacob A. 2022. *Jtools: Analysis and Presentation of Social Scientific Data*. <https://cran.r-project.org/package=jtools>.

Lüdecke, Daniel. 2018a. “Ggeffects: Tidy Data Frames of Marginal Effects from Regression Models.” *Journal of Open Source Software* 3 (26): 772. <https://doi.org/10.21105/joss.00772>.

———. 2018b. “Sjmisc: Data and Variable Transformation Functions.” *Journal of Open Source Software* 3 (26): 754. <https://doi.org/10.21105/joss.00754>.

———. 2022a. *Sjlabelled: Labelled Data Utility Functions (Version 1.2.0)*. <https://doi.org/10.5281/zenodo.1249215>.

———. 2022b. *sjPlot: Data Visualization for Statistics in Social Science*. [https://CRAN.R-project.org/package=sjPlot](https://cran.r-project.org/package=sjPlot).

Lüdecke, Daniel, Mattan S. Ben-Shachar, Indrajeet Patil, Philip Waggoner, and Dominique Makowski. 2021. “performance: An R Package for Assessment, Comparison and Testing of Statistical Models.” *Journal of Open Source Software* 6 (60): 3139. <https://doi.org/10.21105/joss.03139>.

Lüdecke, Daniel, Indrajeet Patil, Mattan S. Ben-Shachar, Brenton M. Wiernik, Philip Waggoner, and Dominique Makowski. 2021. “see: An R Package for Visualizing Statistical Models.” *Journal of Open Source Software* 6 (64): 3393. <https://doi.org/10.21105/joss.03393>.

Müller, Kirill, and Lorenz Walthert. 2022. *Styler: Non-Invasive Pretty Printing of r Code*. [https://CRAN.R-project.org/package=styler](https://cran.r-project.org/package=styler).

O’Hara-Wild, Mitchell, Rob Hyndman, and Earo Wang. 2022a. *Fable: Forecasting Models for Tidy Time Series*. [https://CRAN.R-project.org/package=fable](https://cran.r-project.org/package=fable).

———. 2022b. *Feasts: Feature Extraction and Statistics for Time Series*. [https://CRAN.R-project.org/package=feasts](https://cran.r-project.org/package=feasts).

Pedersen, Thomas Lin. 2022. *Patchwork: The Composer of Plots*. [https://CRAN.R-project.org/package=patchwork](https://cran.r-project.org/package=patchwork).

Poncet, Paul. 2019. *Modeest: Mode Estimation*. [https://CRAN.R-project.org/package=modeest](https://cran.r-project.org/package=modeest).

R Core Team. 2022. *R: A Language and Environment for Statistical Computing*. Vienna, Austria: R Foundation for Statistical Computing. [https://www.R-project.org/](https://www.r-project.org/).

Rodríguez-Sánchez, Francisco, Connor P. Jackson, and Shaurita D. Hutchins. 2022. *Grateful: Facilitate Citation of r Packages*. <https://github.com/Pakillo/grateful>.

Ronnegard, Lars, Xia Shen, and Moudud Alam. 2010. “Hglm: A Package for Fitting Hierarchical Generalized Linear Models.” *The R Journal* 2 (2): 20–28. <https://journal.r-project.org/archive/2010-2/RJournal_2010-2_Roennegaard~et~al.pdf>.

Sievert, Carson. 2020. *Interactive Web-Based Data Visualization with r, Plotly, and Shiny*. Chapman; Hall/CRC. [https://plotly-r.com](https://plotly-r.com/).

Taylor, Sean, and Ben Letham. 2021. *Prophet: Automatic Forecasting Procedure*. [https://CRAN.R-project.org/package=prophet](https://cran.r-project.org/package=prophet).

Wang, Earo, Dianne Cook, and Rob J Hyndman. 2020. “A New Tidy Data Structure to Support Exploration and Modeling of Temporal Data.” *Journal of Computational and Graphical Statistics* 29 (3): 466–78. <https://doi.org/10.1080/10618600.2019.1695624>.

Wickham, Hadley. 2007. “Reshaping Data with the reshape Package.” *Journal of Statistical Software* 21 (12): 1–20. <http://www.jstatsoft.org/v21/i12/>.

Wickham, Hadley, Mara Averick, Jennifer Bryan, Winston Chang, Lucy D’Agostino McGowan, Romain François, Garrett Grolemund, et al. 2019. “Welcome to the tidyverse.” *Journal of Open Source Software* 4 (43): 1686. <https://doi.org/10.21105/joss.01686>.

Wood, S. N. 2017. *Generalized Additive Models: An Introduction with r*. 2nd ed. Chapman; Hall/CRC.

Wood, S. N. 2003. “Thin-Plate Regression Splines.” *Journal of the Royal Statistical Society (B)* 65 (1): 95–114.

———. 2004. “Stable and Efficient Multiple Smoothing Parameter Estimation for Generalized Additive Models.” *Journal of the American Statistical Association* 99 (467): 673–86.

———. 2011. “Fast Stable Restricted Maximum Likelihood and Marginal Likelihood Estimation of Semiparametric Generalized Linear Models.” *Journal of the Royal Statistical Society (B)* 73 (1): 3–36.

Wood, S. N., N., Pya, and B. S"afken. 2016. “Smoothing Parameter and Model Selection for General Smooth Models (with Discussion).” *Journal of the American Statistical Association* 111: 1548–75.

Xie, Yihui. 2014. “Knitr: A Comprehensive Tool for Reproducible Research in R.” In *Implementing Reproducible Computational Research*, edited by Victoria Stodden, Friedrich Leisch, and Roger D. Peng. Chapman; Hall/CRC. <http://www.crcpress.com/product/isbn/9781466561595>.

———. 2015. *Dynamic Documents with R and Knitr*. 2nd ed. Boca Raton, Florida: Chapman; Hall/CRC. <https://yihui.org/knitr/>.

———. 2022. *Knitr: A General-Purpose Package for Dynamic Report Generation in r*. <https://yihui.org/knitr/>.

Xie, Yihui, J. J. Allaire, and Garrett Grolemund. 2018. *R Markdown: The Definitive Guide*. Boca Raton, Florida: Chapman; Hall/CRC. <https://bookdown.org/yihui/rmarkdown>.

Xie, Yihui, Christophe Dervieux, and Emily Riederer. 2020. *R Markdown Cookbook*. Boca Raton, Florida: Chapman; Hall/CRC. <https://bookdown.org/yihui/rmarkdown-cookbook>.

Yutani, Hiroaki. 2022. *Gghighlight: Highlight Lines and Points in ’Ggplot2’*. [https://CRAN.R-project.org/package=gghighlight](https://cran.r-project.org/package=gghighlight).

Zeileis, Achim. 2006. “Implementing a Class of Structural Change Tests: An Econometric Computing Approach.” *Computational Statistics & Data Analysis* 50 (11): 2987–3008. <https://doi.org/10.1016/j.csda.2005.07.001>.

Zeileis, Achim, and Gabor Grothendieck. 2005. “Zoo: S3 Infrastructure for Regular and Irregular Time Series.” *Journal of Statistical Software* 14 (6): 1–27. <https://doi.org/10.18637/jss.v014.i06>.

Zeileis, Achim, Christian Kleiber, Walter Krämer, and Kurt Hornik. 2003. “Testing and Dating of Structural Changes in Practice.” *Computational Statistics & Data Analysis* 44 (1–2): 109–23. <https://doi.org/10.1016/S0167-9473(03)00030-6>.

Zeileis, Achim, Friedrich Leisch, Kurt Hornik, and Christian Kleiber. 2002. “Strucchange: An r Package for Testing for Structural Change in Linear Regression Models.” *Journal of Statistical Software* 7 (2): 1–38. <https://doi.org/10.18637/jss.v007.i02>.
